# The truncated form of flagellin (tFlic) provides the 2dCap subunit vaccine with better immunogenicity and protective effects in mice

**DOI:** 10.1186/s44149-022-00043-x

**Published:** 2022-06-02

**Authors:** Ying Lu, Zehui Liu, Yingxiang Li, Zhuofan Deng, Weihuan Fang, Fang He

**Affiliations:** Department of Veterinary Medicine, Institute of Preventive Veterinary Medicine & Zhejiang Provincial Key Laboratory of Preventive Veterinary Medicine, College of Animal Sciences, Zhejiang University, 866 Yuhangtang road, Hangzhou, 310058 China

**Keywords:** Porcine circovirus type 2 (PCV2), Virus-like particles (VLPs), Truncated form of flagellin (tFlic), Immunogenicity

## Abstract

Porcine circovirus type 2 (PCV2) is the main causative agent of porcine circovirus-associated diseases, and it causes substantial economic losses in the swine industry each year. It is crucial to develop an effective vaccine against the circulating strain PCV2d, which is prone to substantial degrees of mutation. In this study, a truncated form of flagellin (tFlic: 85-111 aa) was inserted into the C-terminal sequence of 2dCap, and Western blotting results showed that recombinant Cap-tFlic VLPs were successfully expressed. Transmission electron microscopy (TEM) and dynamic light scattering (DLS) data indicated that purified recombinant Cap-tFlic fusion proteins existed in the form of polymers and that tFlic could not affect the formation and internalization of VLPs. Integrated Cap-tFlic VLPs induced the expression of antigen presentation-related factors (MHC-II and CD86) by bone marrow-derived dendritic cells (BM-DCs), and the expression of TLR5-related factors (TNF-α) was dramatically elevated. Mice intramuscularly immunized with Cap-tFlic VLPs exhibited significantly higher levels of Cap-specific antibodies and neutralizing antibodies than mice immunized with wild-type Cap VLPs. The data obtained in the current study indicate that Cap-tFlic may be a candidate for a subunit vaccine against PCV2 in the future.

## Main text

Flagellin, the main protein component of bacterial flagella, possesses powerful and modifiable adjuvant activity due to its unique structure. As a pathogen-associated molecular pattern (PAMP) that activates innate and adaptive immune responses, flagellin has been widely used as a potent adjuvant in vaccine design (Khim et al., 2021; Oh et al., 2014; Wangkahart et al., 2018; Gries et al., 2019). Flagellin is the only protein component recognized by the TLR5 receptor that activates NF-κB and stimulates tumor necrosis factor-α (TNF-α) production through a MyD88-dependent pathway (Miao et al., 2007; Benedikz et al., 2019). The innate immune response to bacterial flagellin is mediated by TLR5. Following invasion by a foreign microbe, DCs undergo a maturation process characterized by increased surface expression of MHC-II and costimulatory molecules, and then, effective immune responses in naive T cells are initiated; these phenomena have been confirmed with human dendritic cells (DCs) (Eaves-Pyles et al., 2001; Gewirtz et al., 2004; Means et al., 2003). However, it was shown that murine DCs do not respond to flagellin, and these results may have occurred because these cells do not express TLR5 (Macpherson and Harris, 2004; Uematsu and Akira, 2009). Previous research conducted recent years used flagellin in the development of tumor vaccines, and one such study demonstrated enhanced tumor-specific CD8 ^+^  T cell-mediated immune responses after TLR5 stimulation in a therapeutic cancer vaccine model (Nguyen et al., 2013). It has been shown that flagellin exerts strong adjuvant effects in many vaccine candidates against *Yersinia pestis* (Honko et al., 2006; Mizel et al., 2009), *Plasmodium falciparum* (Bargieri et al., 2008), *Clostridium tetani* (Lee et al., 2006), influenza (Skountzou et al., 2010) and West Nile virus (McDonald et al., 2007). The truncated form of flagellin (tFlic, aa 85-111) and eight other flagellin-related peptides were proven to function as adjuvants and enhance antigen-specific immunity (Faham and Altin, 2010).

Porcine circovirus type 2 (PCV2) was found to cause porcine circovirus-associated diseases (PCVADs). The capsid protein (Cap), encoded by ORF2, is the most important protective antigen of PCV2 and has been used as a target for vaccine development (Chae, 2012). Cap can self-assemble to form virus-like particles (VLPs) of 60 subunits (Khayat et al., 2011). These VLPs have a structure similarity to that of virions that elicits strong B cell-mediated responses and cytotoxic T lymphocyte-mediated responses, but they lack nucleic acids, which reduce the risk of viral spread; thus, VLPs are a hot topic in vaccine development research (Aguilera et al., 2017; Chae, 2016; Fachinger et al., 2008; Rosano and Ceccarelli, 2014; Yin et al., 2010). Epidemiological findings from an increasing number of studies revealed that PCV2d (a PCV2b mutant with reportedly higher virulence) has emerged and spread rapidly in Chinese swine herds (Xiao et al., 2015). Commercial vaccines, including whole-virus inactivated vaccines and recombinant vaccines based on Cap, are in urgent need of improvement.

The structure of VLPs is similar to that of natural viruses; these particles are small and can display repetitive and ordered antigens. It has been demonstrated that VLPs can bind to DCs and be efficiently internalized, promoting stronger MHC-I cross-presentation and T and B cell-mediated responses than soluble antigens (Wu et al., 2010). The self-adjuvant nature of VLPs is considered a prominent advantage, and VLPs are used in vaccine design (Eaves-Pyles et al., 2001). Previous studies have shown that recombinant Cap from PCV2 can be assembled into VLPs in *E. coli*, baculovirus or yeast systems (Bucarey et al., 2009; Marcekova et al., 2009; Nainys et al., 2014). PCV2 Cap was used as a foreign epitope carrier because its C-terminus allows the insertion of small antigen fragments, and the display of small molecules on the surface does not interfere with particle assembly (Li et al., 2018c; Ding et al., 2019; Li et al., 2018a, b). VLPs formed by PCV2 Cap have also been successfully used as foreign epitope vectors to carry other antigens (Zhang et al., 2014). When the nuclear localization signal (NLS) of Cap was replaced with T cell epitopes and B cell epitopes of classical swine fever virus (CSFV), the VLPs could successfully self-assemble and stimulate the production of antibodies against PCV2 and CSFV (Zhang et al., 2014). Furthermore, a recombinant form of PCV2b Cap that includes the VP1 epitope of foot-and-mouth disease virus (FMDV) can form VLPs and elicit strong humoral immunity against PCV2b and FMDV in mice and guinea pigs (Li et al., 2018c). M2e (a conserved protective antigen of the influenza A virus) was expressed in *Escherichia coli* (*E. coli)* and then inserted into the PCV2 Cap sequence, the recombinant Cap self-assembled into chimeric Cap-3M2e VLPs. This bivalent nanovaccine was able to elicit high levels of M2e-specific antibodies and PCV2-specific neutralizing antibodies (Ding et al., 2019). To control the mixed infection of PCV2 and porcine respiratory and reproductive syndrome virus (PRRSV), PCV2-derived VLPs were engineered by replacing the decoy epitope (169-180 aa) of Cap with protective epitopes of PRRSV; these VLPs stimulated the production of considerable amounts of neutralizing antibodies against both PCV2 and PRRSV (Jung et al., 2020). These findings emphasized that genetic recombination of PCV2-derived VLPs is a promising strategy.

In the present study, we explored whether the potency of Cap VLP-based vaccines in a mouse model would be further elevated via the addition of a truncated form of flagellin (tFlic) as an adjuvant.

### Successful expression of Cap-tFlic and assembly of VLPs

**Fig. 1:**
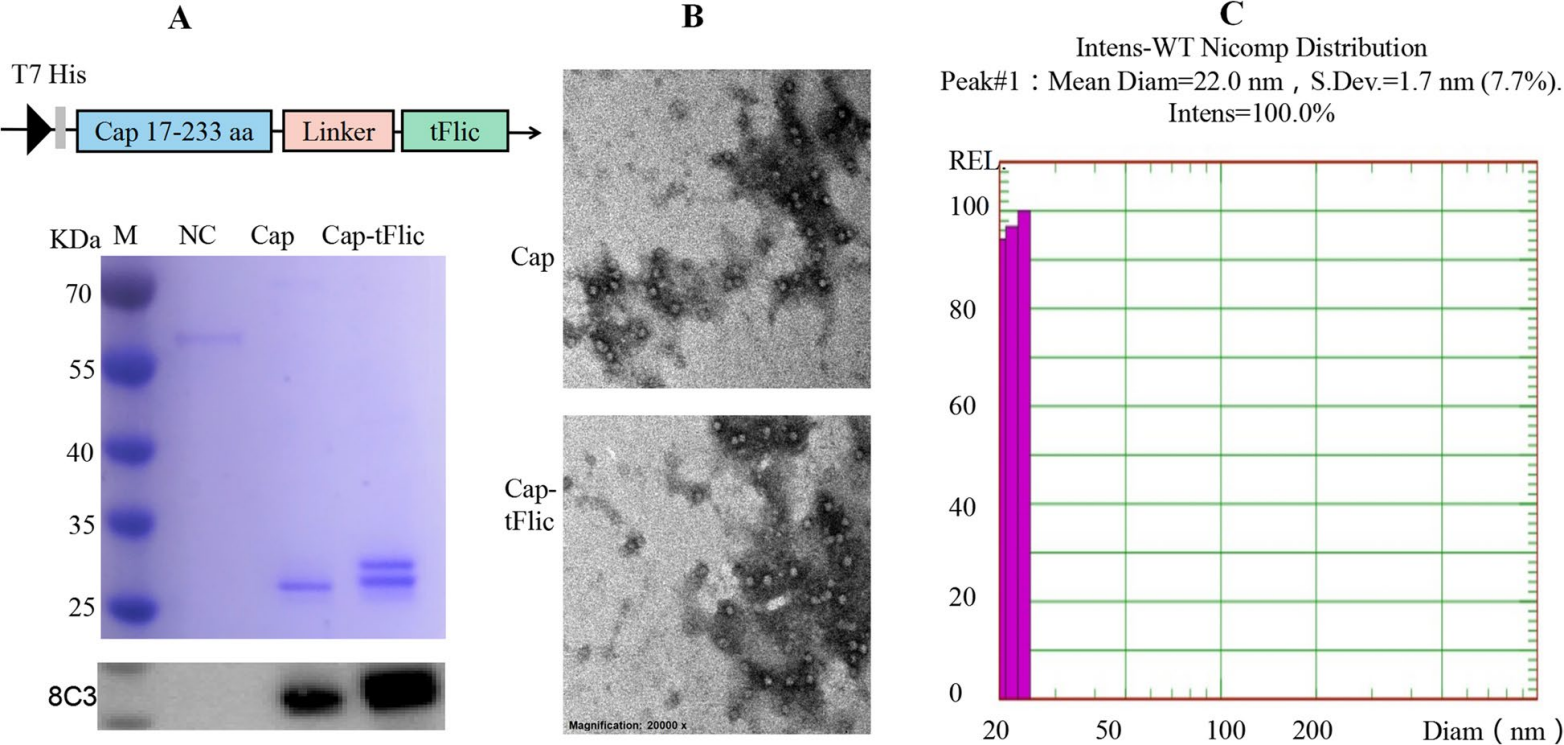
Construction and characterization of Cap-tFlic VLPs. Amino acid sequences corresponding to tFlic were inserted into the C-terminal sequence of Cap, and recombinant proteins were specifically identified by a mouse anti-Cap mAb (8C3) using Western blotting (**A**). Morphological characterization of Cap VLPs by TEM (20,000 ×) (**B**). Determination of Cap-tFlic VLP particle diameter by DLS (**C**). NC, negative control, protein 60 T, ~ 60 kDa

In a previous study, a truncated form of flagellin (tflic, amino acids 85–111 of mature flagellin), which plays an auxiliary role in eliciting immune responses mainly by inducing DC maturation (as shown by MHC-II and CD86 expression) and cytokine production (Liu et al., 2020; Sanders et al., 2009), was used for the targeted delivery of liposomal antigen to APCs in vitro and in vivo; this approach significantly enhanced antigen-specific immunity. To promote the soluble expression of the recombinant protein, amino acids 1-16 of the N-terminus of Cap were deleted. Then, the amino acid sequence of tFlic was inserted into the C-terminus of 2dCap to construct the expression plasmid (Fig. 1A), and Cap-tFlic was transformed into *E. coli* BL21 (DE3) and purified by nickel-nitrilotriacetic acid (Ni–NTA). Western blotting results indicated that the size of the fusion protein was consistent with the expected molecular weight of ~ 28 kDa; moreover, the Cap-tFlic proteins showed significant reactivity with the PCV2 Cap monoclonal antibody 8C3 (Fig. 1A). The purified proteins were processed and analyzed by transmission electron microscopy (TEM). The results showed that Cap-tFlic successfully assembled into regular VLPs with a diameter of ~ 20 nm (Fig. 1B). The DLS results indicated that the major particle size of Cap-tFlic VLPs was 22 nm (Fig. 1C). The observed size of Cap-tFlic VLPs obtained via both methods was consistent, indicating that the Cap-tFlic VLPs exhibit considerable integrity and ordered distribution.

### Significantly elevated antigen-presenting molecule expression via Cap-tFlic VLPs

**Fig. 2:**
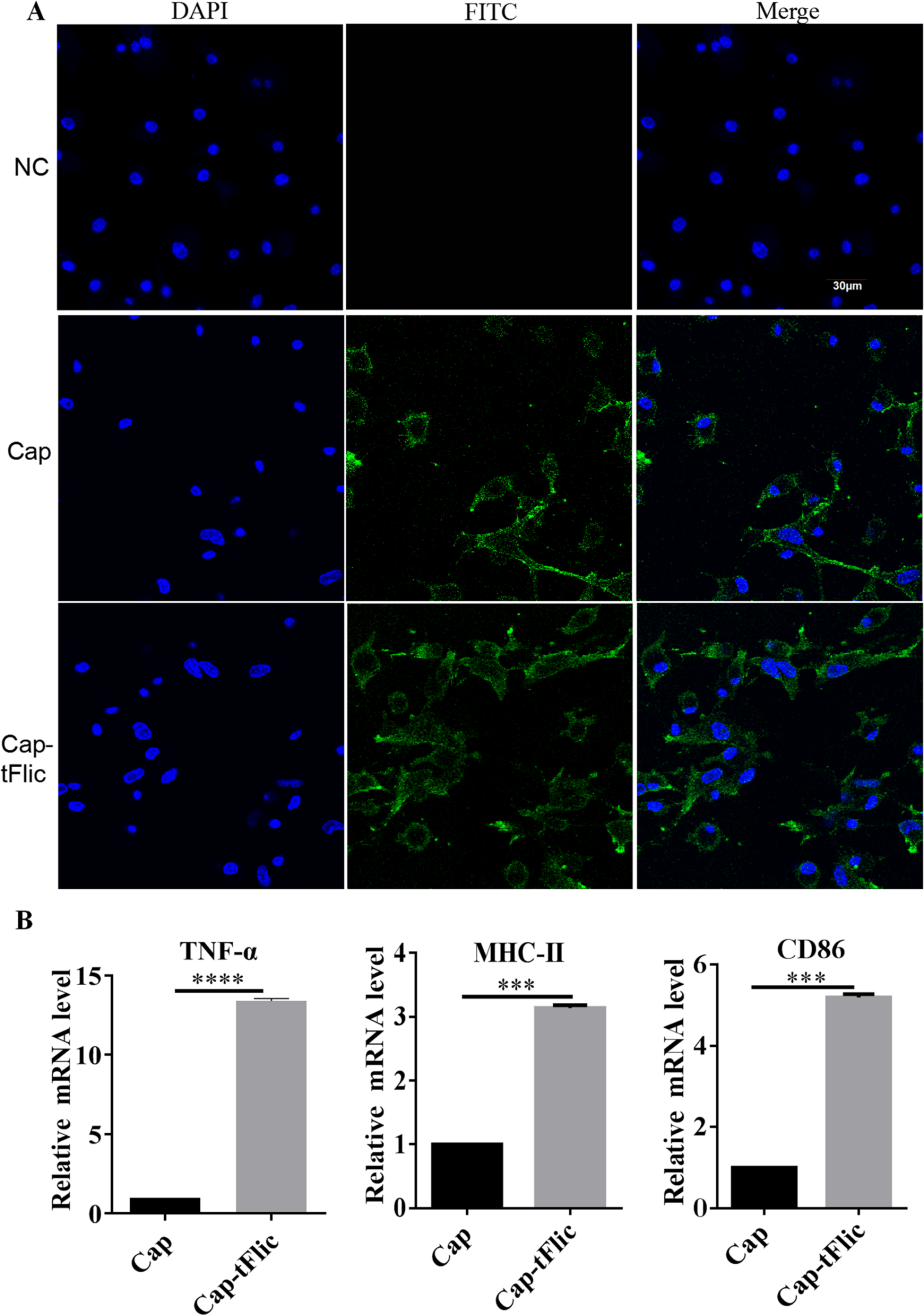
Assessment of antigen uptake and immunostimulatory effects in BM-DCs. BM-DCs were incubated with Cap-tFlic VLPs. The primary antibody was mouse 8C3 mAb, and the secondary antibody was FITC-coupled goat anti-mouse IgG. Signals of cell nuclei (blue) and Cap (green) were detected by CLSM (**A**). BM-DCs were exposed to 100 μg/mL antigens for 12 h, and TNF-α, MHC-II and CD86 expression was then measured by qPCR (**B**). NC, unprocessed BM-DCs. **P < 0.01, ***P < 0.001, ****P < 0.0001

BM-DCs were prepared from mice and used to determine whether the incorporation of antigen with the small molecule adjuvant tFlic influences the uptake of Cap VLPs by APCs. BM-DCs were incubated with 100 μg/mL Cap VLPs and Cap-tFlic VLPs for 12 h. As shown in Fig. 2A, the same green fluorescence signal was observed in the BM-DCs in the Cap VLPs and Cap-tFlic VLPs groups. These results indicate that the ability of the cells to internalize Cap-tFlic VLPs was not enhanced, and conversely, tFlic does not affect the internalization of Cap antigens. DC activation is considered a critical step in initiating adaptive immunity (Banchereau and Steinman, 1998); in fact, upon administration, flagellin induces a strong upregulation of costimulatory molecule expression by DCs (Didierlaurent et al., 2004), and DC activation is usually evaluated by administering potential stimuli and measuring the expression of MHC-II, CD80, CD86 or CD40 (Sanders et al., 2009). To further investigate the initiation of cellular immune responses, relative expression of MHC-II, TNF-α and CD86 was determined by quantitative real-time PCR (qPCR) (Fig. 2B). The results showed that expression levels of MHC-II, CD86 and especially TNF-α markedly increased upon incubation with Cap-tFlic. The level of TNF-α in the Cap-tFlic VLP group was 13.39-fold higher than that in the native Cap VLP group. The levels of MHC-II and CD86 in the Cap-tFlic VLP group were 3.14- and 5.18-fold higher than those in the native Cap VLP group, respectively. At present, most of studies related to flagellin that used mouse models mostly used intestinal or lung-derived monocytes. Interestingly, when mouse-derived DCs were stimulated by Cap VLPs carrying flagellin (flagellin was the only variable) in this study, the expression of TLR5-related cytokines, such as TNF-α, was increased; however, the mechanism underlying tFlic-induced changes in the expression of relevant factors requires further study. These results suggest that Cap-tFlic VLPs can induce cytokine production and DC maturation in vitro.

### Stimulation of potent humoral immune responses by Cap-tFlic VLPs

**Fig. 3:**
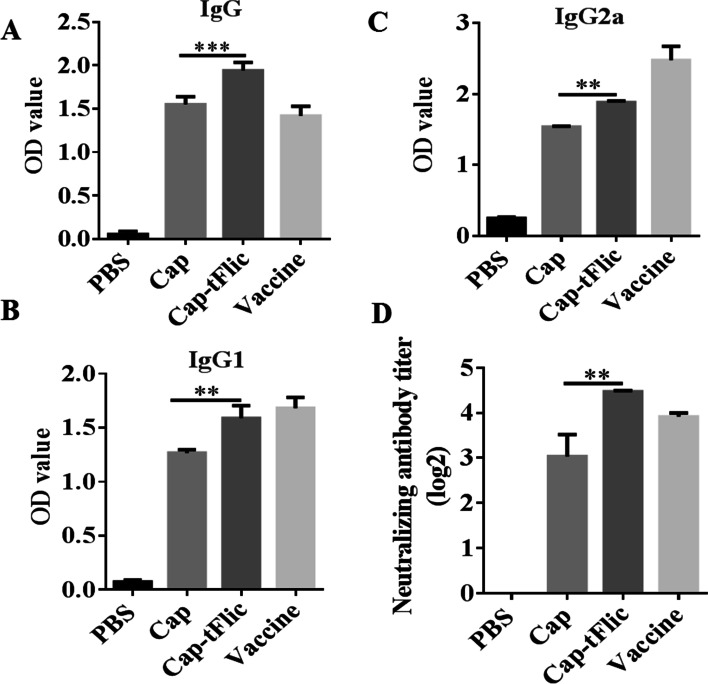
Measurement of Cap-specific antibody levels and PCV2-specific Nab levels in sera. Levels of specific antibodies, including IgG (**A**), IgG1 (**B**) and IgG2a (**C**), and neutralizing antibodies against the PCV2 JH strain were measured by ELISA at 35 dpi (day post immunization). NAb titers were calculated and are expressed as the log 2 of the reciprocal of the highest serum dilution that was able to completely block PCV2 infection in PK-15 cells (**D**). **, *P* < 0.01, ***, *P* < 0.001

After immunization of a mouse model, it was observed that the efficient internalization of Cap-tFlic indeed correlated with a more effective humoral immune response, especially with a significant increase in IgG1 and IgG2a production. IgG2a production suggests a predominantly T helper cell type 1 (Th1) response, which plays an indispensable role in protection against viral infection (Hannestad and Scott, 2017). Therefore, the levels of anti-Cap antibodies were tested by enzyme-linked immunosorbent assay (ELISA). The results are shown in Fig. 3. As expected, insignificant antibody responses were observed in the negative control group that was treated with PBS, and Cap-specific antibody levels were significantly elevated in the immunized group. At 35 d post immunization (dpi), the level of IgG detected in the antigen-immunized Cap VLP group was higher than that in the positive control group, and the Cap-tFlic VLP group has significantly higher IgG levels than the Cap VLP group (*p* < 0.001), corresponding to the OD450 value in the Cap-tFlic VLP group being 1.25-fold higher than that in the Cap VLP group. The distribution of the IgG subclasses IgG1 and IgG2a was then assessed. The IgG1 levels of the Cap-tFlic VLP group were significantly higher than those of the Cap VLP group (*P* < 0.01), corresponding to the OD450 value in the Cap-tFlic VLP group being 1.25- and 1.22-fold higher than that in the Cap VLP group. Levels of virus-neutralizing antibodies (NAbs) against PCV2 correlate well with in vivo protection; they can bind to antigens on the surface of pathogenic microorganisms, preventing adherence to target cell receptors and cell invasion. Therefore, the titer of NAbs in serum samples was measured (Fig. 3). As expected, no PCV2-specific NAbs were detected in the negative control group, and significant increases in the NAb levels were detected in all the other immunized groups. In the Cap-tFlic VLP groups, the average NAb titer was 1:20.53, which was significantly (*P* < 0.01) higher than that in the native Cap VLP groups (1:10.13). These results suggest that Cap-tFlic VLPs are more efficient in eliciting a Th1 response and humoral immune responses than conventional Cap VLPs in vivo, verifying that the addition of the truncated form of flagellin (tFlic) in the PCV2 subunit vaccine represents an alternative strategy for improving the immunogenicity of Cap.

## Methods

### Cells, virus and experimental animals

The GenBank accession number of the Cap gene is MT376345.1, and the amino acid sequence of the truncated form of flagellin (tFlic: 85-111 aa) is ATCAACAACAACCTGCAGCGTGTTCGTGAACTGGCTGTTCAGTCTGCTAACTCTACCAACTCTCAGTCTGACCTGGACTCT. Porcine kidney epithelial cells (PK-15, ATCC -33,BNCC, China) were cultured in Dulbecco’s modified Eagle’s medium (DMEM, Thermo, USA) supplemented with 10% fetal bovine serum (FBS, Yeasen, China), 100 IU/mL penicillin (Sangon, China) and 100 mg/L streptomycin (Sangon, China) at 37 °C in a 5% CO_2_ atmosphere. The PCV2d JH strain (MG_245867.1) was grown in PK-15 cells and utilized for indirect immunofluorescence assay (IFA) and ELISA. Totally 20 eight-week-old female SPF BALB/c mice and C576BL male mice were arbitrarily chosen and purchased from the Experimental Animal Center of Zhengjiang University. The experimental mice were randomly divided into four groups, given clean water and food, and allowed to acclimate to the housing environmental conditions. The animal experimental procedures were supervised and approved by the Laboratory Animal Management Committee, Zhejiang University.

### Cloning and expression of Cap-tFlic VLPs

As shown in Fig. 1, the small molecule adjuvant truncated flagellin (tFlic) was fused to the N-terminus of Cap with a 4 × GGS flexible linker to generate the recombinant plasmid pET28a-2dcap-d16-tFlic, which was then transformed into *E. coli* Rosetta (DE3) (Yeasen, China) competent cells. The sequences of the primers used for PCR are shown in Table 1. Bacteria were cultured, treated with 0.06 mM isopropyl thiogalactoside (IPTG, Sangon, China), collected, suspended and lysed by ultrasonication. Then, supernatants containing the target proteins were collected and mixed with agarose resin (Yeasen, China), and target proteins were eluted with PBS supplemented with 500 mM imidazole (Liu et al., 2021). For the subsequent experiments, the purified proteins were analyzed by SDS–PAGE and Western blotting.

**Table 1:** The primers used for PCR

Primer	Sequence (5′-3′)
tFlic-F	TCAGTCTGACCTGGACTCTTGAGATCCGGCTGCTAACAAAGCCCG
tFlic-R	AACACGCTGCAGGTTGTTGTTGATACCACCCTTAGGGTTAAGTGGGGGGTC
pET28a-2dCap-d16-tFlic-F	TGATGAAAGCTTGGCACTGGCCGTCGTTTTACAACGTCG
pET28a-2dCap-d16-tFlic-R	CACTTTGTGATTCATATGCTATGGTCCTTGTTGGTGAAG
β-actin-F	GGAGGGGGTTGAGGTGTT
β-actin-R	GTGTGCACTTTTATTGGTCTCAA
MHC-II-F	CTGTCTGGATGCTTCCTGAGTTT
MHC-II-R	TCAGCTATGTTTTGCAGTCCACC
TNF-α-F	GCCTCTTCTCATTCCTGCTT
TNF-α-R	TGGGAACTTCTCATCCCTTTG
CD86-F	GCCGTGCCCATTTACAAAGGCTCAA
CD86-R	TGTTACATTCTGAGCCAGTTTTATT

### Characterization of Cap-tFlic particles

Purified Cap-tFlic fusion proteins were filtered and dialyzed at 16 °C overnight as described previously (Liu et al., 2021). VLPs were visualized by transmission electron microscopy (TEM, JEOL, JEM-1010) using 2% phosphotungstic acid (PTA, Aladdin, China) (pH 6.8) negative staining. The size distribution and diameter of the VLPs were then measured by dynamic light scattering (DLS, PSS, Nicomp, USA.)

### Culture of murine bone marrow-derived dendritic cells (BM-DCs)

BM-DCs were isolated and cultured as described previously (Liu et al., 2021). Briefly, C576BL male mice were euthanized and dissected, and bone marrow-derived dendritic cells were resuspended and seeded in 6-well cell plates at a density of 10^6^ cells/well. Then, the cells were cultured for 7 d in complete RPMI-1640 (Thermo,USA) medium supplemented with 10 ng/mL granulocyte–macrophage colony-stimulating factor (GM-CSF, Sangon, China) and 10 ng/mL interleukin-4 (IL-4, Sangon, China).

### Cellular uptake

Internalization of Cap VLPs by BM-DCs was measured as described previously (Liu et al., 2021). In brief, 100 μg/mL Cap VLPs and Cap-tFlic VLPs were added to cell cultures and incubated for 12 h. The cells were fixed with cold acetone, blocked with FBS, incubated with an anti-Cap monoclonal antibody (8C3), which was previously identified by this laboratory, for 1 h, and incubated with a fluorescent secondary antibody. The cells were stained with 4,6-diamino-2-phenyl indole (DAPI, Abcam, China) for 10 min, and then, the immunofluorescence signals of Cap internalization were measured by confocal laser scanning microscopy (CLSM, Japan, Olympus, IX81-FV1000).

### Analysis of cytokine mRNA expression in BM-DCs

The effect of Cap VLPs on BM-DC antigen presentation was investigated as described previously (Liu et al., 2021). After exposure to 100 μg/mL Cap VLPs or Cap-tFlic VLPs for 12 h, total RNA was isolated from BM-DCs with an Easy RNA Kit (Easydo, China), and mRNA expression was analyzed by quantitative real-time PCR (qPCR). The primers used for qPCR are shown in Table 1.

### Immunization

Eight-week-old female BALB/c mice (Zhejiang University Animal Experiment Center) were randomly divided into four groups with five animals per group, and the mice were subcutaneously inoculated with 4 μg of Cap VLPs or Cap-tFlic VLPs. PBS and commercial subunit vaccine (Pulike, China) were used as negative and positive controls, respectively. Booster immunization was carried out with the same dose at 14 dpi, and serum samples were collected to measure the levels of PCV2-specific antibodies.

### Serological assays

Mouse serum samples were collected at 35 dpi and were subjected to indirect ELISA to measure Cap-specific antibody levels as described previously (Liu et al., 2021). Briefly, 0.5 μM/mL 2dCap was precoated overnight at 4 °C, blocked, incubated with mouse serum samples (diluted in PBST, 1:500) for 1 h at 37 °C, and incubated with HRP-conjugated goat anti-mouse IgG/IgG1/IgG2a (BBI, Beijing) diluted in PBST by 1:5000, 1:2000 and 1:2000. Then, tetramethylbenzidine (TMB, Invitrogen, USA) was added, and the absorbance at 450 nm was read by a microplate reader (Biotek, Epoch, USA).

To determine the titers of neutralizing antibodies (NAbs) against PCV2, a virus neutralization test (VNT) was performed as described previously (Lu et al., 2022). In brief, PK-15 cells were cultured in DMEM a confluence of 70%, and twofold serially diluted heat-inactivated serum samples were mixed with the same volume of PCV JH (200 TCID_50_) and incubated for 1 h at 37 °C. The cells were incubated with the mixture for 1 h and then incubated under normal conditions for 72 h. Then, IFA was performed, and the cells were observed under a fluorescence microscope.

### Statistical analysis

Student’s t test was used to analyze the significance of the differences between two groups. *P* values < 0.05 were considered statistically significant.

## Data Availability

The data will be shared upon request by readers.

## Abbreviations

tFlic: Truncated form of flagellin
PCV2: Porcine circovirus type 2
VLPs: Virus-like particles
DLS: Dynamic light scattering
MHC-I: Major histocompatibility complex class I
MHC-II: Major histocompatibility complex class II
TNF-α: Tumor necrosis factor-α
PAMP: Pathogen-associated molecular pattern
TLR5: Toll-like receptor
DCs: Dendritic cells
Cap: Capsid protein
*E. coli*: Escherichia coli
NLS: Nuclear localization signal
FMDV: Foot-and-mouth disease virus
CSFV: Classical swine fever virus
PRRSV: Porcine respiratory and reproductive syndrome virus
Ni–NTA: Nickel-nitrilotriacetic acid
PBS: Phosphate-buffered saline
PBST: Phosphate Tween-20 buffer
TEM: Transmission electron microscopy
BM-DCs: Bone marrow-derived dendritic cells
qPCR: Quantitative real-time PCR
NAbs: Neutralizing antibodies
PMWS: Postweaning multisystemic wasting syndrome
NK: Natural killer cells
IL-6: Interleukin-6
IL-10: Interleukin-10
Th1: T helper cell type 1
PK-15: Porcine kidney epithelial cells
DMEM: Dulbecco’s Modified Eagle Medium
FBS: Fetal bovine serum
IFA: Indirect immunofluorescence assay
ELISA: Enzyme-linked immunosorbent assay
IPTG: Isopropyl thiogalactoside
PTA: Phosphotungstic acid
GM-CSF: Granulocyte–macrophage colony-stimulating factor
IL-4: Interleukin-4
CLSM: Confocal laser scanning microscopy
DAPI: 4,6-Diamino-2-phenyl indole
TMB: Tetramethylbenzidine
VNT: Virus neutralization test
